# Search for evolutionary roots of land plant arabinogalactan-proteins in charophytes: presence of a rhamnogalactan-protein in *Spirogyra pratensis* (Zygnematophyceae)

**DOI:** 10.1111/tpj.15577

**Published:** 2021-11-26

**Authors:** Lukas Pfeifer, Jon Utermöhlen, Kathrin Happ, Charlotte Permann, Andreas Holzinger, Klaus von Schwartzenberg, Birgit Classen

**Affiliations:** 1 Department of Pharmaceutical Biology, Pharmaceutical Institute, Christian-Albrechts-University of Kiel, Kiel 24118, Germany; 2 Department of Botany, Functional Plant Biology, University of Innsbruck, Innsbruck 6020, Austria; 3 Biocenter Klein Flottbek, University of Hamburg, Hamburg 22609, Germany

**Keywords:** arabinogalactan-proteins, charophyte algae, cell wall, plant evolution, *Spirogyra pratensis*, *Nitellopsis obtuse*, Charophyceae, Zygnematophyceae, polysaccharides, terrestrialization, Yariv’s reagent

## Abstract

Charophyte green algae (CGA) are assigned to be the closest relatives of land plants and therefore enlighten processes in the colonization of terrestrial habitats. For the transition from water to land, plants needed significant physiological and structural changes, as well as with regard to cell wall composition. Sequential extraction of cell walls of *Nitellopsis obtusa* (Charophyceae) and *Spirogyra pratensis* (Zygnematophyceae) offered a comparative overview on cell wall composition of late branching CGA. Because arabinogalactan-proteins (AGPs) are considered common for all land plant cell walls, we were interested in whether these special glycoproteins are present in CGA. Therefore, we investigated both species with regard to characteristic features of AGPs. In the cell wall of *Nitellopsis*, no hydroxyproline was present and no AGP was precipitable with the β-glucosyl Yariv’s reagent (βGlcY). By contrast, βGlcY precipitation of the water-soluble cell wall fraction of *Spirogyra* yielded a glycoprotein fraction rich in hydroxyproline, indicating the presence of AGPs. Putative AGPs in the cell walls of non-conjugating *Spirogyra* filaments, especially in the area of transverse walls, were detected by staining with βGlcY. Labelling increased strongly in generative growth stages, especially during zygospore development. Investigations of the fine structure of the glycan part of βGlcY-precipitated molecules revealed that the galactan backbone resembled that of AGPs with 1,3-1,6- and 1,3,6-linked Gal*p* moieties. Araf was present only in small amounts and the terminating sugars consisted predominantly of pyranosidic terminal and 1,3-linked rhamnose residues. We introduce the term ‘rhamnogalactan-protein’ for this special AGP-modification present in *S. pratensis*.

## Introduction

Plant cells are surrounded by a cell wall rich in polysaccharides. In most cases, cellulose microfibrils build a framework associated with diverse types of polysaccharides and glycoproteins. Cell walls are dynamic structures that are constantly modified during development or in response to biotic or abiotic stress.

A major group of cell wall proteins are hydroxyprolinerich glycoproteins (HPRGs), which comprise the three subgroups proline-rich proteins, extensins and arabinogalactan-proteins (AGPs) (Johnson et al., 2018). The proline-rich proteins are only minimally glycosylated, whereas the glycan part of extensins is around 50% and consists of single galactose residues attached to serine and arabinose-oligosaccharides attached to hydroxyproline (Hyp). In AGPs, the polysaccharide part is dominating (around 90%) with the main monosaccharides arabinose (Ara) and galactose (Gal). These arabinogalactans are covalently linked to the protein moiety via Hyp and are characterized by a typical galactan core structure consisting of a backbone of 1,3-linked β-d-Gal*p*, branched at position 6 to 1,6-linked β-d-Gal*p* side chains, which are substituted with α-l-Ara*f* and often also terminal Glc*p*A. Furthermore, a typical feature of AGPs is their ability to precipitate with Yariv phenylglycosides, such as the β-glucosyl Yariv’s reagent (βGlcY) (Ma et al., 2018). For some AGPs, linkage to the plasma membrane by a glycosylphosphatidylinositol anchor has been demonstrated (Youl et al., 1998), which makes these molecules ideal candidates for communication between the cytoplasm and the extracellular matrix. The involvement of AGPs in different processes such as cell growth, cell proliferation, pattern formation and reproduction has been demonstrated (Ma et al., 2018; Seifert and Roberts, 2007) and it is generally accepted that AGPs are important signaling molecules, although the mechanism of action is still unclear. Currently, knowledge of the structure of AGPs in plants, especially in the angiosperms, is extensive and, in seed plants, it appears that these molecules are ubiquitous. The occurrence of AGPs in spore-producing land plants was already shown in the 1970s via gel-diffusion assays with Yariv’s reagent. On the other hand, the structural characterization of AGPs from these plants is still limited (Classen et al., 2019). The first investigations revealed that AGPs from ferns, lycophytes and mosses not only possess the general structural elements known from seed plants, but also show unique features, possibly involved in diverse functions. High amounts of terminal pyranosidic arabinose have been detected in the AGP of the lycophyte *Lycopodium* (Bartels and Classen, 2017) and the unusual monosaccharide 3-*O*-methylrhamnose is present in moss and fern AGPs (Bartels et al., 2017; Fu et al., 2007) and also some gymnosperms (Baumann et al., 2021).

The question of whether AGPs evolved in response to the demands of the terrestrial environment or whether they already existed in the algal ancestors is currently a focus of AGP research. Approximately 500 million years ago (mya), land plants evolved from a freshwater alga of the charophyte lineage and, by the end of the Devonian (360 mya), the extant lineages of land plants (liverworts, hornworts, mosses, lycophytes, monilophytes and spermatophytes) arose and today they dominate the terrestrial environment (Bowman et al., 2017; de Vries and Archibald, 2018; Delwiche and Cooper, 2015; Harrison, 2017). The transition from water to land required molecular adaptations to cope with an array of new stresses (e.g. desiccation and UV irradiation). It is reasonable to assume that the conquest of land required severe changes in cell wall composition (Harholt et al., 2016; Sørensen et al., 2011). Bioinformatic as well as biochemical search for glycosyl-transferases in different charophyte green algae (CGA) revealed evidence that land plant cell wall biosynthetic mechanisms are present within them (Herburger et al., 2019; Mikkelsen et al., 2014, 2021). AGPs are considered common for all land plant cell walls and are involved in different processes such as tolerance to drought stress or to other abiotic and biotic stress situations (Mareri et al., 2018) and they therefore comprise molecular candidates important for the drastic habitat change during terrestrialization. Furthermore, the involvement of AGPs during embryogenesis (Coimbra et al., 2007) and in plant–microbe interactions (Nguema-Ona et al., 2013) has been shown for many land plant species and implicates that AGPs might also play a role in evolution of these processes. According to bioinformatic screening based on large-scale transcriptomic data for high numbers of plant species, protein backbones of AGPs have been proven for members of the brown, red and green algae (Johnson et al., 2017) and analysis of the *Nostoc punctiforme* genome identified five putative AGP core proteins even in the cyanobacterium *Nostoc* (Jackson et al., 2012). Nevertheless, when and how the glycan structures of AGPs evolved is still questionable. Using monoclonal antibodies directed against oligosaccharide epitopes, AG-epitopes in different algae have been detected in brown algae (Hervé et al., 2016; Raimundo et al., 2016), in chlorophyte green algae (Estevez et al., 2008, 2009; Přerovská et al., 2021) and also in charophyte algae (Domozych et al., 2009; Eder et al., 2008; Palacio-López et al., 2019; Sørensen et al., 2011). It needs to be taken into account that the exact epitopes of these antibodies are often unknown and also show cross-reactivities with other polysaccharides (e.g. pectins). For a definite proof of AGPs in land plant ancestors and detection of structural differences, the isolation and structural characterization of these molecules from the different groups of algae is necessary.

The closest relatives to land plants belong to the group of CGA, which are divided into the lower-branching KCM-grade (Klebsormidiophyceae, Chlorokybophyceae and Mesostigmatophyceae) and the higher-branching ZCC-grade (Zygnematophyceae, Coleochaetophyceae and Charophyceae) (de Vries and Archibald, 2018). Several CGA are used as model organisms to investigate plant development and stress physiology, and the simple thalli (many of them stay unicellular) facilitate experimental manipulation (Domozych et al., 2016). In the past, morphologically complex forms of the Charophyceae such as *Chara* species have been considered as sister group of land plants. However, recent phylogenomic analyses revealed that land plants evolved from streptophyte algae most closely related to extant Zygnematophyceae (Leebens-Mack et al., 2019; Ruhfel et al., 2014; Timme et al., 2012; Wickett et al., 2014; Wodniok et al., 2011). To the best of our knowledge, no pure AGP has been isolated from any charophyte alga to date. We hypothesized that algae with the closest relationship to land plants will contain AGPs with structural similarities to land plant AGPs. We decided to use the morphologically complex species *Nitellopsis obtusa*, which is a member of the Charophyceae and *Spirogyra pratensis* and an established model organism of the Zygnematophyceae (Domozych et al., 2016; Zhou and von Schwartzenberg, 2020), to search for evolutionary roots of these glycoproteins. Microscopic investigations of conjugating *Spirogyra* after staining with βGlcY complemented the analytical investigations. Our results revealed fundamental differences of the cell walls of *Nitellopsis* and *Spirogyra* that support the sister group relationship between land plants and the Zygnematophyceae. The presence of evolutionary ancestor molecules of AGPs in charophytes enlightens molecular adaptions of these glycoproteins with regard to plant terrestrialization.

## Results

### Sequential extraction of polysaccharide fractions from charophyte cell walls

**Figure 1:**
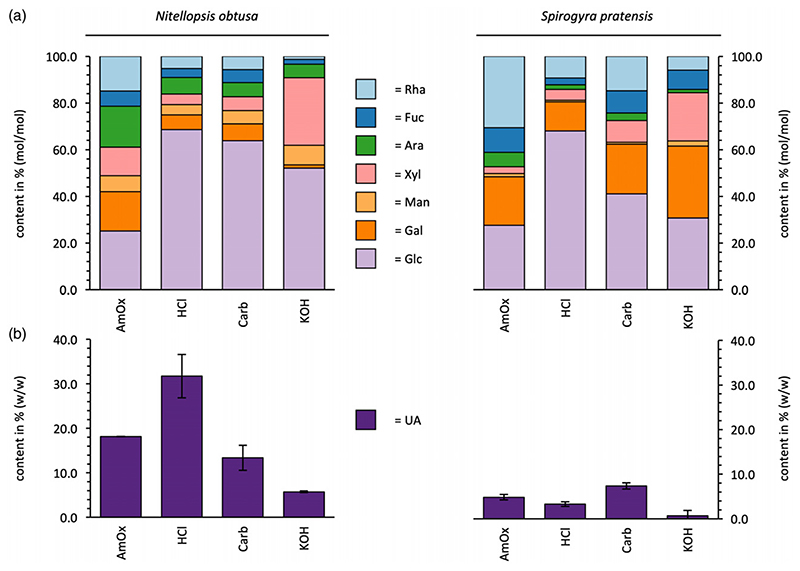
Carbohydrate composition of the extracts from *Nitellopsis obtusa* and *Spirogyra pratensis*. (a) Neutral monosacccharide composition determined by gas chromatography (% mol/mol). (b) Absolute content of uronic acids determined by colorimetric assay (% w/w of dry plant weight)

Extraction with different solvents resulted in cell wall fractions enriched in specific polysaccharides. Two pectic fractions soluble in acidic solvents (ammonium oxalate and dilute hydrochloric acid) and two alkali-soluble (sodium carbonate and dilute potassium hydroxide) hemicellulose fractions were isolated. Yields of the different fractions are given in Figure S1. The monosaccharide composition varied between the fractions and the two species (Figure 1; Table S1a–d).

Similar trends in both algae were identified: the fractions isolated with ammonium oxalate contained more rhamnose (Rha) and Ara compared to the other fractions, the fractions isolated with HCl were very rich in glucose (Glc) and the fractions isolated by KOH contained highest amounts of xylose (Xyl). Furthermore, appreciable amounts of fucose (Fuc) were present in all fractions.

In *Nitellopsis*, both fractions isolated with acidic solvents were dominated by higher amounts of uronic acids (UA) compared to the other fractions, indicating the presence of pectic polysaccharides. Further monosaccharides that often occur in pectic polysaccharides, such as Rha, Gal, Ara and Xyl, were also found in these fractions. The essential difference between the ammonium oxalate and the hydrochloric acid fraction was the amount of Glc, which was considerably higher in the HCl fraction. Glc also dominated the Na_2_CO_3_ and KOH fractions. The composition of the carbonate fraction was comparable to the HCl fraction with lower amounts of UA, whereas the KOH fraction was clearly dominated by Glc and Xyl (together more than 80%, mol/mol).

In *Spirogyra*, the fractions isolated by acidic solvents clearly differed compared to *Nitellopsis* as a result of their low amounts of UA, atypical for pectic polysaccharides. The ammonium oxalate fraction was very rich in Rha, Glc and Gal, accompanied by Fuc and Ara and low amounts of Xyl and Man. Comparable to *Nitellopsis*, the HCl fraction contained almost 70% (mol/mol) of Glc. Main monosaccharides of the alkaline fractions were Glc, Gal, Rha, Fuc and Xyl (carbonate fraction) and Glc, Gal, Xyl and Fuc (KOH fraction). The Fuc content in both fractions was noticeable. In all fractions of *Spirogyra*, the Rha content was doubled or more than doubled compared to the same fractions of *Nitellopsis*. Furthermore, all fractions of *Spirogyra* were richer in Gal and contain less Ara and Man compared to the fractions of *Nitellopsis*.

### Isolation and characterization of high-molecular weight, water-soluble fractions (AE) from cell walls of *Nitellopsis* and *Spirogyra* as a potential source of AGPs

High molecular weight fractions were isolated by aqueous extraction followed by ethanol precipitation. The yield of this fraction for *Nitellopsis* was 5.0% (w/w) in relation to the dry weight of algal material and 8.1 and 7.2% (w/w) for two different batches of *Spirogyra*, respectively.

#### Monosaccharide composition of aqueous extracts (AE)

The water-soluble and ethanol-precipitated fraction of *Nitellopsis* (AE) was mainly composed of the monosaccharides Glc, Ara, Gal and Rha and was further purified by tangential cross-flow filtration (AE_purified_), which led to reduction of the amount of Glc (Table 1). Because the aqueous fraction from *Spirogyra* differed strongly compared to *Nitellopsis*, a second batch was investigated. Both batches of *Spirogyra* were comparable with striking dominance of Rha and Gal, accompanied by appreciable amounts of Fuc and Ara, as well as low amounts of Xyl, Man, Glc, methylated Rha and methylated Gal. UA were present in amounts of 3.8% (w/w, *Nitellopsis*) and 5.0% (w/w, *Spirogyra*, batch 1), respectively.

#### Protein and Hyp content of aqueous extracts (AE)

Elemental analysis was performed and nitrogen content used for estimation of protein amount. The water-soluble high molecular weight fractions of *Nitellopsis* and *Spirogyra* contained 1.7% (w/w) and 2.1% (w/w) of nitrogen, respectively, accounting for 10.6% (w/w) and 13.1% (w/w) of protein according to the Kjeldahl method. Tangential cross flow filtration of *Nitellopsis* AE reduced the amount of nitrogen to 1.1% (w/w) accounting for 6.9% (w/w) of protein in AE_purified._

In AGPs, the glycan moieties are bound to the protein via *O*-glycosidic linkage to the amino acid Hyp. Therefore, the absolute amount of Hyp in both AE samples was determined by a photometric method (Stegemann and Stalder, 1967) (detection limit 0.004%, w/w) and found to be 0.45% (w/w) for *Spirogyra*, whereas no Hyp could be detected in *Nitellopsis*. Furthermore, none of the other extracts of *Nitellopsis* contained any Hyp.

#### Gel diffusion assay

A gel diffusion assay with βGlcY was used to test the occurrence of AGPs in AE of both species (Figure S2). A red precipitation band occurred with AE of *Spirogyra* but was missing in *Nitellopsis*, indicating the presence of AGPs only in *Spirogyra*.

### Isolation and characterization of the Yariv fraction (YF) from the water-soluble fraction of *Spirogyra*

As expected based on the previous results (lack of Hyp, no precipitation band in gel diffusion assay) no βGlcY precipitation was obtained from the water-soluble fraction of *Nitellopsis*. By contrast, Yariv precipitation of *Spirogyra* aqueous fraction yielded 0.71% (w/w, batch 1) and 0.87% (w/w, batch 2) material relative to dry weight of algal material.

#### Monosaccharide composition of YF

The monosaccharide composition of the fraction precipitated with Yariv’s reagent was very unusual for an AGP because Ara was present only in low amounts (Table 2). Instead, Gal and Rha strongly dominated and were accompanied by lower amounts of Fuc and Ara. Glc, Man and Xyl were present in very small portions between 1 and 2% (mol/mol). The composition of both batches was comparable. The photometric assay detected 8.0% (w/w) of UA in this fraction. Because UA are not detectable in the acetylation analysis followed by GC, reduction of UA to the corresponding neutral monosaccharide was performed prior to analysis (YF_UR). GlcA was detected as deuterium-labelled Glc. Furthermore, a new peak occured in YF_UR and was identified as 4-*O*-methyl-glucuronic acid (4-*O*MeGlcA). Further methylated monosaccharides were also part of the Yariv-precipitated material and identified as 2-*O*-methyl-rhamnose (2-*O*MeRha), 3-*O*-methyl-rhamnose (3-*O*MeRha) and 3-*O*-methyl-galactose (3-*O*MeGal).

#### Protein and Hyp content of YF

The amount of nitrogen in this material was 1.8% (w/w), corresponding to 11.3% (w/w) of protein according to the Kjeldahl method. Hyp was quantified photometrically and accounted for 0.57% (w/w) of the precipitated material, which means that Hyp accounts for 5.1% (w/w) of the protein.

#### Linkage analyses of YF from *Spirogyra* in comparison to purified aqueous extract from *Nitellopsis*

No βGlcY-precipitable fraction was present in *Nitellopsis*. Therefore, the ethanol-precipitated aqueous extract was further purified by tangential cross flow filtration with a molecular weight cut-off (MWCO) of 50 kDa (AE_purified_). This fraction was also subjected to linkage analysis (Table S2). The main linkage types known for seed plant AGPs were present in high amounts: terminal Ara*f* (15.8%), 1,5-linked Ara*f* (14.6%), 1,3-linked Gal*p* (13.5%), 1,6-linked Gal*p* (12.1%) and 1,3,6-linked Gal*p* (11.3%). These arabinogalactan moieties were mainly accompanied by 1,4-linked Rha*p* (Table S2).

*Spirogyra* YF was subjected to linkage analysis after carboxy-reduction and also after additional partial acid hydrolysis (Table 3). The investigations confirmed that YF was not a typical AGP with mainly Gal and Ara but consisted mainly of Gal and Rha. Linkage types of Gal were identical to AGPs from land plants with terminal, 1,3-, 1,6- and 1,3,6-linked Gal*p*. Instead of Ara*f*, which was detected only in very small amounts, the terminal residues were mainly Rhap. Interestingly, the other linkage type of Rha was 1,3-Rhap, which is unusual for plant polysaccharides but was clearly identified by fragmentation in MS (Figure S3). Additionally, terminal GlcA*p* was found in minor amounts. The primary fragment (*m/z* 207) (for deuterated terminal Glc) was accompanied by only traces of the corresponding non-deuterated fragment (*m/z* 205), indicating that only terminal GlcA and not Glc is present in the native YF. Neutral monosaccharide analysis (Table 2) revealed that this terminal GlcA is methylated at O-4. Deuterium-labelled Glc was also present in 1,4-linkage. Small amounts of 1,6-linked Man*p* might be part of *N*-glycans known from chimeric AGPs (Strasser, 2016) and have therefore not been included in the proposed structure (see Discussion).

Mild acid hydrolysis (YF_UR_hydr_) led to complete loss of terminal Ara*f*, Fuc*p* and GlcA*p*, as well as a slight reduction of terminal Rha*p* and 1,4-linked Glc/GlcA*p*. Table S3 illustrates the changes of the galactan framework of YF_UR during partial hydrolysis. The decrease of 1,3,6-linked Gal*p* corresponded to the increase of terminal and 1,6-linked Gal*p*, suggesting that Rha*p* is mainly attached to positions 3 and 6 of 1,3,6-Gal*p* in the native AGP. Loss of terminal 4-*O*MeGlcA*p* and Fuc probably converted 1,6-Gal to terminal Gal*p*.

### ELISA with antibodies detecting AG-epitopes

**Figure 2:**
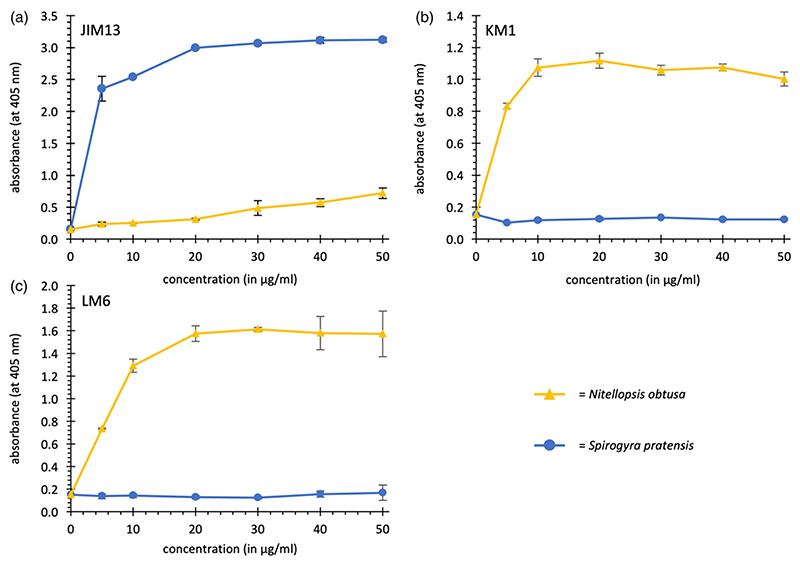
Reactivity of *Nitellopsis obtusa* AE_purified_ and *Spirogyra pratensis* Yariv fraction with antibodies directed against arabinogalactan-protein glycans in an ELISA. (a) JIM13. (b) KM1. (c) LM6

Three antibodies against AG-epitopes were tested for interaction with AE from *Nitellopsis* and the YF from *Spirogyra* (Figure 2a–c). JIM13 is known to bind to many AGPs but the exact epitope is under debate. The trisaccharide β-d-GlcA*p*(1 → 3)-α-d-GalA*p*-(1 → 2)-α-l-Rha*f* binds to this anti-body (Yates et al., 1996). KM1 has been raised against *Echinacea* AGP (Classen et al., 2004) and β-d-1,6-Gal*p* is part of the epitope (Bartetzko et al., 2015; Ruprecht et al., 2017). LM6 is directed against α-l-1,5-linked Ara*f* (Verhertbruggen et al., 2009).

JIM13 showed low reactivity with *N*. *obtusa* AE and stronger with KM1 and LM6. Binding affinities of *S*. *pratensis* YF were completely different with stronger binding to JIM13 and no binding to KM1 and LM6. The detected binding affinities supported the results of linkage analyses: 1,6-linked Gal*p* (KM1) and 1,5-linked Ara*f* (LM6) are present in *N*. *obtusa* AE in amounts of 12.1 and 14.6%, respectively. The strong interaction of *S*. pratensis YF with JIM13 reflects that Rha and GlcA might be important monosaccharides of the epitope of this antibody. JIM13 and KM1 were also tested with the partially hydrolysed YF from *S*. *pratensis* (Figure S4a,b). With JIM13, the reactivity persisted, and with KM1, reactivity increased as a result of an increase of 1,6-Gal*p* after mild acid hydrolysis.

Summing up, a comparison of the different AGP features present or absent in *Nitellopsis* and *Spirogyra* is presented in Table 4. Binding to antibodies directed against AG-epitopes was opposed in cell walls of both species. Although the AE fraction of *Nitellopsis* was rich in Ara and Gal and contained protein, no Hyp was present and no precipitation with Yariv’s reagent was possible. By contrast, AE of *Spirogyra* was rich in Gal and Rha but poor in Ara, and contained Hyp and precipitated with Yariv’s reagent. We therefore introduce the term ‘rhamnogalactan-protein’ for these glycoproteins present in *S. pratensis*.

### Microscopic detection of *Spirogyra* rhamnogalactan-proteins (RGPs) with βGlcY

Although interaction of AGPs with Yariv’s reagent is still not fully understood, it is known that mainly the galactan part of AGPs is responsible for binding, and especially 1,3-linked Gal oligosaccharides are essential (Kitazawa et al., 2013). Our analytical results reveal that in case of *Spirogyra*, the stained molecules are RGPs.

After treatment with βGlcY, staining in non-conjugating, fast-growing vegetative filaments of *S. pratensis* mainly appeared in transverse walls (Figure S5, arrows).

**Figure 3:**
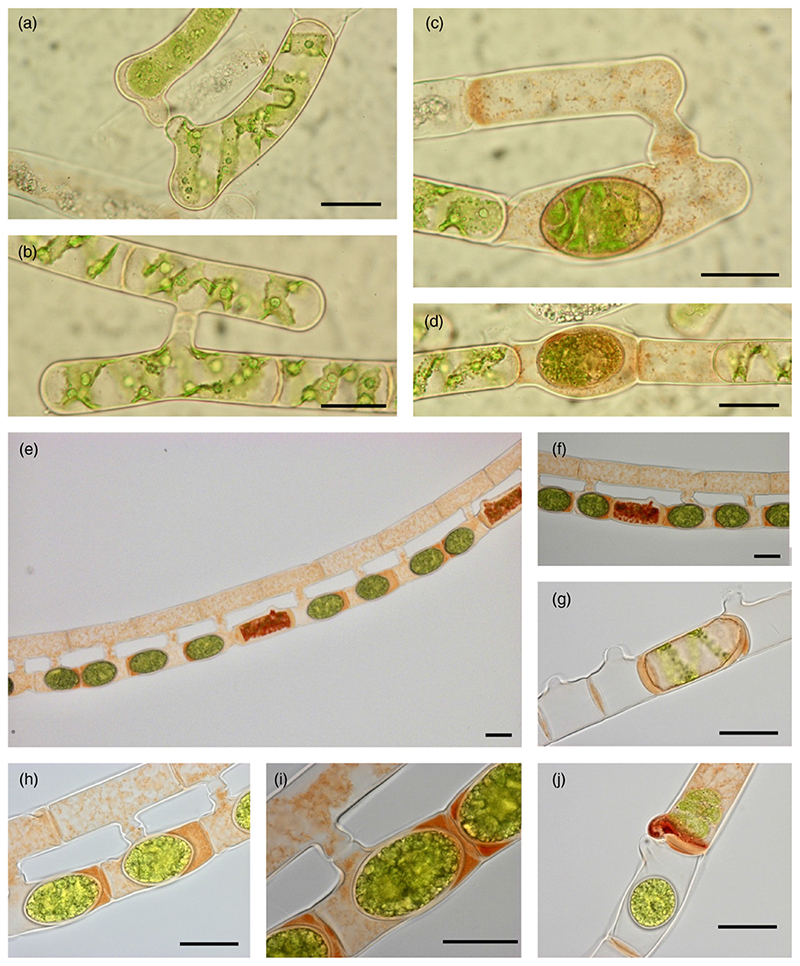
Conjugation and zygospores of *Spirogyra pratensis* and *Spirogyra* sp., staining with βGlcY. (a–d) *Spirogyra pratensis*. (e–j) *Spirogyra* sp. field sample from Kühtai, Tyrol, Austria (a,b) Early stages of scalariform conjugation with weak β-glucosyl Yariv’s reagent (βGlcY) staining. (c–j) Stages with zygospores. (c) Scalariform conjugation with βGlcY staining in conjugation tube, donor and recipient cell, in the latter surrounding the zygospore. (d) Lateral conjugation with βGlcY staining in the donor and recipient cell, in the latter surrounding the zygospore. (e,f) Scalariform conjugation with zygospore formation in the recipient filament. Both donor cells and cells with zygospores are filled with βGlcY-positive material. In the recipient filament, single cells without zygospores and small papillae are stained strongly. (g) Scalariform conjugation with staining of transverse walls and one cell completely surrounded by a βGlcY-positive wall. (h,i) Scalariform conjugation with zygospore formation in the recipient filament. Both donor cells and cells with zygospores are filled with βGlcY-positive material, sometimes concentrated adjacent to one side of the zygospore. (j) Lateral conjugation where the papilla of the donor cell is strongly stained. Scale bars = 20 µm.

Furthermore, two *Spirogyra* species with conjugation stages were investigated (Figure S6; Figure 3). In *S. pratensis*, conjugation was successfully induced under laboratory conditions (Zhou and von Schwartzenberg, 2020), whereas *Spirogyra* sp. was field-sampled. In Figure S6, unstained algae or algae treated with the negative control αGalY are shown. No staining was observed with the negative control (Figure S6a–h) compared to the untreated algae (Figure S6i–k). Mature zygospore walls are red-colored in both species (Figure S6e,g,k). Figure 3 presents both algae after positive staining with βGlcY. In both species, both lateral and scalariform conjugation appeared, the latter with a much higher frequency. Scalariform conjugation is characterized by a papilla first in one filament (Figure S6a,b) and then another filament lying in the adjacent position, thus forming a conjugation tube (Figures 3a,b). Lateral conjugation occurs in one filament, starting with bulges emerging on both sides of a transverse wall (Figure S6c,d) and growth of a conjugation tube. The protoplast of the donor cell migrates to the recipient cell and syngamic fusion leads to formation of a diploid zygote, which develops to a zygospore with a thick cell wall (Figure S6e–k; Figures 3c–f, h–j). In scalariform conjugation, zygospores mostly occurred in one filament, whereas zygospore formation in both filaments was only occasionally observed.

Both species showed strong staining with βGlcY during the conjugation process (Figure 3). After zygote formation, staining of both species indicated RGPs surrounding the zygospores and also filling the ‘empty’ donor cell (Figure 3c–f,h–i). This was observed not only in scalariform, but also in lateral conjugation (Figure 3d). Staining around zygospores was sometimes uneven (Figure 3h,i) or even missing (Figure 3j). In lateral conjugation of *Spirogyra*. sp., strong staining of the papilla of the donor cell was sometimes observed (Figure 3j). Only in *Spirogyra* sp., single cells without conjugation partner between others with zygospores showed only a small papilla and were strongly stained, indicating the massive presence of RGPs in the whole cytoplasm (Figures 3e,f). Furthermore, whole cells not involved in conjugation were sometimes completely surrounded by a RGP-rich wall (Figure 3g).

## Discussion

Approximately 500 mya, an ancestral taxon of the CGA class Zygnematophyceae performed the successful transition from water to land, which led to global radiation of land plants and finally to the Earth’s diverse terrestrial ecosystems. The success of these pioneering land plants was dependent on key attributes, and plant evolutionary biology aims to uncover the features of such algae that enabled this important transition (de Vries and Archibald, 2018). One prerequisite to performing terrestrialization was the ability to produce a cell wall with a particular composition (Sørensen et al., 2011).

Based on transcriptomes of *Coleochaete* and *Spirogyra*, it has been stated that ‘the charophyte green algal transcriptomes are remarkably plant-like’ (Timme and Delwiche, 2010). With regard to cell walls, bioinformatic search for glycosyltransferases in different CGAs based on transcriptomic data also revealed similarities to land plants (Delaux et al., 2015; Mikkelsen et al., 2014, 2021). Nevertheless, the biosynthesis of cell wall components is still not fully understood. Isolation and structural characterization of cell wall polysaccharides and glycoproteins from different CGAs is necessary to extend our understanding of the cell wall composition of CGAs in comparison to recent descendants of the first land plants.

### Polysaccharides present in cell walls of *Nitellopsis* and *Spirogyra*

#### Pectins

In *Nitellopsis*, the fractions isolated with ammonium oxalate and HCl show a monosaccharide composition typical for pectins. Although the HCl fraction is even richer in UA, Glc is dominating, indicating the presence of glucans in addition to pectin. In *Spirogyra*, no typical pectic fraction was isolated because the amounts of UA in fractions isolated with ammonium oxalate and HCl were low. Investigations using the monoclonal antibodies 2F4, JIM5 and JIM7 directed against homogalacturonan (HG) epitopes revealed the presence of HG in different members of CGA (Domozych et al., 2014; Eder and Lütz-Meindl, 2008, 2010; Herburger et al., 2019; Palacio-López et al., 2019; Proseus and Boyer, 2006), although the glycan microarray with JIM5 and 2F4 also revealed strong differences. There was strong reactivity with cyclohexane-trans-1,2-diamine-*N*,*N*,*N′*,*N′*-tetraacetate (CDTA) extracts of *Chara*, *Coleochaete*, *Cosmarium*, *Penium* and *Netrium*, whereas no reactivity was detected in *Spirogyra*, *Klebsormidium* and *Chlorokybus* (Sørensen et al., 2011). By contrast, CDTA extract of *Spirogyra mirabilis* showed strong binding especially to LM19, recognizing HG with a low degree of esterification, and also good binding to JIM5 and 2F4. Furthermore, antibodies recognizing epitopes of RG I also showed weak interaction with *Spirogyra* extracts (Permann et al., 2021a).

From *Nitella tanslucens* and *Chara australis*, pectins mainly consisting of non-esterified GalA have been purified (Anderson and King, 1961a,b). Isolation of pectins by oxalate followed by paper chromatography verified HG for all investigated CGA except *Klebsormidium* and suggested the presence of RG I only in the later-diverging CGA (O’Rourke et al., 2015). Investigations on glycosyl linkages revealed only low levels of RG I in CGA, and none of the characteristic sugars of RG II (2-*O*MeFuc, 2-*O*MeXyl, apiose, aceric acid) were detected (Sørensen et al., 2011). Future investigations should aim to clarify, whether mainly HG or also RG I and RG II are pectic polysaccharides present in members of the CGA and in which species and amounts they occur.

#### Glucans and callose

Both investigated algae, especially the fractions isolated with HCl, were dominated by Glc, raising the question of ehat type of glucans are present. Because fractions were not treated with amylase, starch might be present. Mixed-linked glucans known from grasses (Gibeaut and Carpita, 1993) and also from *Equisetum* (Fry et al., 2008) have also been detected in different CGA, although they were mainly present in the alkalisoluble fractions (Eder et al., 2008; Sørensen et al., 2011). Callose was not found in cell walls of *Micrasterias* but was present in *Zygnema* (Herburger and Holzinger, 2015) and it is essential for the completion of cytokinesis in *Penium* (Davis et al., 2020).

#### Hemicelluloses

Mannans, xylans and xyloglucans have been found in the cell walls of CGA members (Domozych et al., 2012; Popper and Tuohy, 2010). Both investigated algae and especially *Spirogyra* contained only small amounts of Man, indicating that mannans are not abundant in *Nitellopsis* and *Spirogyra*. By contrast, glycan microarrays with antibody BS-400-4 revealed high amounts of mannans in *S. mirabilis* (Permann, et al., 2021a). The fractions isolated with KOH of both investigated algae contain high amounts of Xyl and Glc, possibly indicating xylans and xyloglucans. Linkage analyses are necessary to confirm the identity of these polysaccharides. The presence of both polysaccharides in *Zygnema* was confirmed by the fact that extracts of this alga exhibited transglycosylase activity towards xyloglucan and xylan (Herburger et al., 2018). Xyloglucan was originally assumed to be absent in CGA (Popper and Fry, 2003), but there is now evidence that xyloglucans evolved in these algae (Mikkelsen et al., 2021) and act as a substrate of unusual charophytic transglycosylases (Franková and Fry, 2021). Evidence for xylans and xyloglucans in CGA is further based on glycan microarrays with antibodies directed against epitopes present in these polysaccharides (Domozych et al., 2009; Eder and Lütz-Meindl, 2008; Ikegaya et al., 2012; Mikkelsen et al., 2021; Permann et al., 2021b; Sørensen et al., 2011), as well as structure elucidation (Ikegaya et al., 2008; Mikkelsen et al., 2021). These analytical data are complemented by studies of biosynthetic enzymes (Del-Bem and Vincentz, 2010; Mikkelsen et al., 2014, 2021; Nishiyama et al., 2018). The cell wall of *Micrasterias* was labelled by a polyclonal anti-xyloglucan antibody but not by the monoclonal antibody CCRC-M1, which recognizes Fuc- and Gal-containing side-chains of xyloglucans (Eder and Lütz-Meindl, 2008). Recent investigations revealed the presence of fucosylated and galactosylated xyloglucans in *Mesotaenium* (Mikkelsen et al., 2021), indicating that these changes of the basic xyloglucan structure are already present at least in some CGA species. Because our investigations revealed appreciable amounts of Fuc and Gal besides Xyl and Glc in the *Spirogyra* fractions isolated with carbonate and KOH, the presence of fucosylated and galactosylated xyloglucans in *S. pratensis* might also be possible. It has been reported that the secretion of xyloglucans allowed early terrestrial CGA to aggregate soil particle around cells (Del-Bem, 2018).

In conclusion, sequential extraction of *Nitellopsis* and *Spirogyra* cell walls support the hypothesis that major polysaccharides known from land plants (pectins, mannans, xylans, xyloglucans) are present in these species. The monosaccharide composition of the pectic polysaccharides differs between both species. That of *Nitellopsis* resembles those of land plants, whereas the pectic fraction of *Spirogyra* is atypical with low amounts of UA. Furthermore, other special features pronounced in *Spirogyra* are the quite high amounts of Rha and Fuc in all fractions, whereas the content of Ara is very low. This is an indication that other polysaccharides might be present or that known polysaccharides are decorated with side chains different from those of land plants.

### Search for AGPs in *Nitellopsis* and *Spirogyra*

To search for AGPs, fractions AE were isolated from *Nitellopsis* and *Spirogyra*, revealing strong differences between both species. AE from *Nitellopsis* showed a mixed monosaccharide composition including Ara and Gal, whereas AE of *Spirogyra* was dominated by Rha and Gal. Especially the Rha content was extraordinarily high, indicating a unique polysaccharide composition, maybe including rhamnans. In different green algae, rhamnan sulfates have been detected (Cui et al., 2019; Lee et al., 2010; Liu et al., 2018). In *Spirogyra neglecta*, aqueous extraction followed by ethanol precipitation yielded a fraction rich in Rha, Fuc, Gal and Glc and poor in Ara, Xyl and Man (Surayot et al., 2015).

#### Protein moiety of *Nitellopsis* AE and *Spirogyra* YF

Bioinformatic screening of transcriptomic data for high numbers of plant and algal species revealed the presence of AGP protein backbones in the CGA (Johnson et al., 2017). Therefore, it was surprising that the βGlcY-precipitated fraction from *Spirogyra* resembled an AGP with regard to protein and Hyp amount, although no Hyp was detected in AE of *Nitellopsis*. Furthermore, none of the other extracts of *Nitellopsis* contained any Hyp. A search for Hyp in several members of green algae confirmed its occurrence in different members of chlorophyte algae and in *Spirogyra* (Zygnematophyceae) but not in *Nitella* (Charophyceae, Gotelli and Cleland, 1968; Morrison et al., 1993; Thompson and Preston, 1967). Further members of the CGA have to be investigated with regard to Hyp content and the presence of prolyl-hydroxylase to confirm or falsify the generalization that Hyp is present in members of the Zygnematophyceae and not in the Charophyceae. It should be taken into account that Hyp is also present in extensins and prolylhydroxylase also acts in the biosynthesis of these members of HPRGs. Because *Nitellopsis* AE contains protein but no Hyp, binding of AG glycans to the protein is theoretically possible by *N*-glycosylation of asparagine (Strasser, 2016) or *O*-glycosylation of serine or threonine. Binding of single Gal residues to Ser is a feature of extensins and linkage of arabinogalactan to threonine has been proposed for radish AGP (Tsumuraya et al., 1987).

#### Carbohydrate moiety of *Nitellopsis* AE and *Spirogyra* YF

*Nitellopsis* AE was very rich in typical AGP linkage types of Ara and Gal. Comparable to *Micrasterias* (Eder and Lütz-Meindl, 2008), there was no interaction with βGlcY, although binding of AGP-antibodies was observed.

βGlcY-precipitated fraction of *Spirogyra* revealed a unique composition with galactan features of a type II AGP but mainly terminal and 1,3-linked rhamnose residues instead of Ara. In *Arabidopsis* cell wall, an AGP-RG Iarabinoxylan complex has been detected (Tan et al., 2013). Because βGlcY-precipitated material of *Spirogyra* contained no GalA (Figure S7a) and no linkage types of Rha typical for RG I (1,2-Rha, 1,2,4-Rha), the presence of an AGP-RG I complex in *Spirogyra* is unlikely. We therefore propose using the term ‘rhamnogalactan-protein’ for this special AGP-modification detected in *S. pratensis*. Terminal GlcA is methylated at C-4, a feature also known from seed plant AGPs (Temple et al., 2019; Tryfona et al., 2012). Further methylated monosaccharides (2-*O*- and 3-*O*MeRha, 3-*O*MeGal) occur in minor amounts, which all have also been identified in polysaccharides from *Chlorella vulgaris* (Ogawa et al., 1994, 1997). 3-*O*MeRha has been found in cell walls of *Chara*, *Coleochaete* and *Klebsormidium* (Popper et al., 2004), in AGPs of mosses and ferns (Bartels et al., 2017; Bartels and Classen, 2017; Fu et al., 2007), and also in gymnosperm AGPs (Baumann et al., 2021), whereas 3-*O*Me-Gal was detected in CGAs and lycophytes, but not in mosses (O’Rourke et al., 2015).

The results of an ELISA with antibodies directed against AG epitopes confirmed the strong differences between both investigated CGAs: *Nitellopsis* AE reacted with KM1 and LM6, whereas *Spirogyra* YF showed strong reaction with JIM13.

The antibodies KM1 and LM6 recognize typical epitopes of AGPs. These are a Gal-epitope with β-d-1,6-linked Gal*p* (KM1) and α-l-1,5-linked Ara*f* (LM6). Both antibodies interacted with the AE of *Nitellopsis* because these linkage types are present although an AGP is missing. This indicates that the interaction with these antibodies is not always a definite proof of the presence of AGPs. JIM13 is an antibody generally regarded to be AGP-specific, although the exact epitope is still unknown. There was strong reaction with the RGP of *Spirogyra*, indicating that especially Rha and maybe GlcA are part of the epitope. The fact that the trisaccharide β-d-GlcA*p*(1 → 3)-α-d-GalA*p*-(1 → 2)-α-Rha*f*, which is rather untypical for AGPs, binds to JIM13 (Yates et al., 1996) confirms this assumption. Different antibodies directed against AG epitopes (LM2, LM14, MAC 207, JIM8, JIM13 and JIM20) have been tested in glycan microarray for interaction with CDTA and NaOH extracts of different CGAs (Sørensen et al., 2011). In that study, JIM13 was the only antibody binding to *Spirogyra* extracts, thus confirming our results. By contrast, all anti-bodies (except for LM2) bound strongly to extracts of *Chara corallina*. *Coleochaete* (Coleochaetophyceae), also a member of the higher-branching CGAs, exclusively reacted with LM2 and MAC207 (Sørensen et al., 2011). These results reveal strong differences between the Charophyceae and the Zygnematophyceae comparable to our investigation. The lower-branching KCM-grade was represented by *Klebsormidium*, which showed no reactivities with these AGP-antibodies; see also Steiner et al. (2020). Other studies investigated the same antibodies and extract types of *Mougeotia* sp. and *S. mirabilis*. The strongest interaction in both cases was also detected with JIM13 (Permann et al., 2021a,b). Because AGP-RG I-complexes have been described for Arabidopsis (Tan et al., 2013), we also tested two antibodies specific for RG I (namely INRA-RU1 and -2). In *Spirogyra*, both antibodies lacked concentration-dependent reactivity (Figure S7b), thus confirming the absence of RG-I epitopes with a minimum two repeats of the disaccharide (-Rha-1,4-GalA-1,2-) (Ralet et al., 2010).

**Figure 4:**
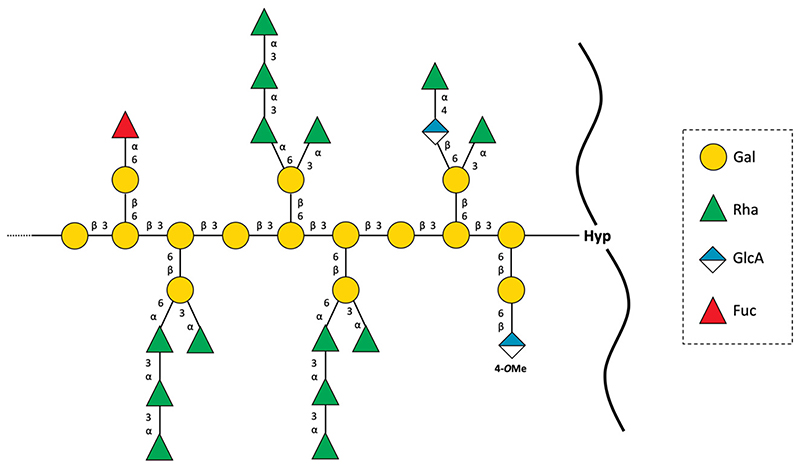
Structural proposal for a rhamnogalactan-protein present in *Spirogyra pratensis* cell wall.

Based on methylation analysis before and after partial hydrolysis and the results of an ELISA, a structural proposal for the RGP from *Spirogyra* is given in Figure 4. Terminal fucosylation is a minor decoration of angiosperm AGPs (Tryfona et al., 2012) and was partially present in *Arabidopsis* AGP via α-1,2-linkages on Ara*f* residues (Tryfona et al., 2012). Because 1,2-linked Ara*f* was detected only in traces in *Spirogyra* RGP, it was not included in the structural proposal. Substitution of the β-1,6-galactan side chains with 4-*O*MeGlcA at their non-reducing termini is also a feature known from angiosperm AGPs (Jiao et al., 2017; Tryfona et al., 2012), as well as the motif terminal Rha-(1 → 4)-GlcA-(1 → 6)-Gal (Tan et al., 2010).

### Microscopic localization of RGPs in *Spirogyra*

Monoclonal antibodies directed against AG-epitopes are tools for the microscopic detection of AGPs in algae. In CGA, AG-epitopes were detected by JIM8 and JIM13 in *Chara* (Domozych et al., 2009), by JIM8, JIM13 and JIM14 in *Micrasterias* (Eder et al., 2008; Lütz-Meindl and Brosch-Salomon, 2000; Sørensen et al., 2011), by JIM13 in *Mougeotia* and *Spirogyra* (Permann et al., 2021a,b), and by JIM8 and JIM13 in *Chlorokybus*, *Coleochate*, *Penium*, *Zygnema* and *Spirogyra* (Domozych, 2019; Palacio-López et al., 2019). JIM8 is recognizing an epitope containing 1,6-linked Gal (Ruprecht et al., 2017), but our results on *Nitellopsis* have shown that this epitope is present, although AGPs are missing. The exact epitopes of JIM13 and JIM14 are unknown and therefore binding to other polysaccharides (e.g. to side chains of pectins) might be possible. In case of *Spirogyra*, our results confirm that JIM13 recognizes RGPs, not AGPs. The microscopic localization of AGPs is further possible by staining with the red coloured dye βGlcY. A 1,3-linked Gal oligosaccharide was identified to be necessary for binding (Kitazawa et al., 2013; Paulsen et al., 2014). Yariv-staining occurred during *Fucus* early embryogenesis (Hervé et al., 2016), although Ara is absent in the cell walls (Raimundo et al., 2016), thus supporting our results that RGPs of *Spirogyra* (possibly also present in the field sample of *Spirogyra* sp.) interact with βGlcY and Ara is not essential for binding to this specific dye. Staining of *Spirogyra* rhizoids by βGlcY (Palacio-López et al., 2019) is probably also a result of the presence of RGPs.

Conjugation of *Spirogyra* has been investigated in filaments from algae collections (e.g. Microalgae and Zygnematophyceae Collection Hamburg) or from native habitats (Permann et al., 2021a), often after induction of conjugation in the laboratory (Ikegaya et al., 2012; Takano et al., 2019; Yoon et al., 2009; Zwirn et al., 2013). Especially culture conditions with nitrogen depletion and increased light intensity promote conjugation in the Zygnematophyceae including *S. mirabilis* (Permann et al., 2021a). In *S. pratensis*, conjugation was induced by transferring the cells from liquid medium to agar medium where conjugation occurred spontaneously after several days. Although sexual reproduction is a well-known phenomenon, the underlying molecular mechanisms are still unknown. Labeling of *Spirogyra* with plant lectins during the conjugation process revealed first evidence for involvement of saccharides (Ikegaya et al., 2012; Yoon et al., 2009). *Ricinus communis* agglutinin, which binds Gal, led to a decrease of conjugation in *Spirogyra* (Yoon et al., 2009). In *Mougeotia*, JIM13 detected AGPs (probably RGPs) in the outer spore wall with fibrous appearance (Permann et al., 2021b) and, in *S. mirabilis*, JIM13 labelled the zygospore wall and also produced a signal in conjugating gametangia (Permann et al., 2021a). In both *Spirogyra* species investigated, the cell wall of the zygospore is red-coloured without staining, which makes it difficult to detect further staining by βGlcY. Despite this, strong binding of βGlcY was observed. In both species, βGlcY-precipitated material surrounds the zygospore, filling not only the whole cell, but also the adjacent donor cell, indicating that RGPs are involved in the conjugation process.

A role of AGPs in sexual reproduction of angiosperms is well documented (Ma et al., 2018) and has also been described for the gymnosperm *Larix* (Rafińska et al., 2021) and the fern *Ceratopteris* (Lopez and Renzaglia, 2014, 2016). An ovular AGP named AMOR with the terminal disaccharide 4-*O*-methyl-glucuronosyl galactose (possibly also present in *Spirogyra* RGP) (Figure 4) is involved in pollen tube guidance in *Torenia* (Jiao et al., 2017).

Further investigations on sexual reproduction of spore-producing land plants and algae are necessary to clarify the role of AGPs and their molecular ancestors in this process.

## Conclusions

Based on bioinformatic screenings, protein backbones of AGPs have been confirmed for members of the brown, red and green algae, including different members of CGA. Knowledge on AGP glycosylation is mainly based on search for glycosyltransferases in CGA transcriptomes and glycan microarrays or microscopy with antibodies directed against AG-epitopes. Both approaches have some limitations. First, biosynthesis of the AGP glycan chains is not fully understood and some glycosyltransferases have the ability to act on different polysaccharides. Second, epitopes of antibodies are not always clearly defined and might also be present on other polysaccharides. Based on our results, the idea that AGPs are present in CGA needs to be revised and more CGA species must be investigated to further enlighten the evolutionary process of these glycoproteins. It remains to be determined whether the unique modification with the unusual terminal disaccharide Rha*p*-(1 → 3)-Rha*p* detected in *Spirogyra* is present in other members of the Zygne-matophyceae and plays a role in sexual reproduction. Our results propose the first occurence of AGP (and RGP) specific galactan moieties linked to proteins via Hyp in the most recent common ancestor of Zygnemato-phyceae and embryophytes. Further knowledge on cell walls of the Zygnematophyceae and other CGAs will contribute to a better understanding of early land plant evolution, especially with regard to terrestrialization.

## Experimental Procedures

### Plant material, growth conditions and sampling

*Nitellopsis obtusa* (Desvaux) J.Groves (Charophyceae) was collected in Stechlin-Ruppiner Land Nature Park from Mittlerer Giesenschlagsee by Silke Oldorff.

*Spirogyra pratensis* transeau (Zygnematophyceae) strain MZCH10213 was obtained from the Microalgae and Zygnematophyceae Collection Hamburg (Zhou and von Schwartzenberg, 2020) and was grown under axenic conditions in WHM-medium as described previously (Nichols, 1973), with modifications, at 21 ± 1°C under fluorescent lighting at approximately 80 µmol L−1 photons m−2 sec−1 with a 12:12 h light/dark photocycle. The alga was grown as airlift culture in 2.0-L culture flasks airated with sterile air (approximately 500 mL min−1) for 21 days.

The alga filaments were separated from the culture medium by a sieve (mesh width 100 µm). The remaining culture medium was removed with the help of sterile paper towels. The material of both algae was freeze-dried and stored at −20°C.

To induce conjugation, vegetative filaments grown in liquid WHM-medium were transferred to Petri dishes containing C-medium (Ichimura, 1971) solidified with 1% (w/w) agar. After 8–14 days, conjugation and zygospore formation was observed.

The field sample of conjugating *Spirogyra* sp. was taken in the Austrian alps (Kühtai) in early August 2021 (2020 m.a.s.l./47°21′76″N, 11°03′77″). The sampling site was a small rivulet with a slow velocity in vicinity of the sampling sites of *S. mirabilis* (Permann et al., 2021a). The sample was stored in water taken from the sampling site at 16/8 h, 20/15°C and approximately 30 µmol photons m^−2^ sec^−1^ photosynthetically active radiation. Microscopical investigations were conducted on the following day.

### Isolation of cell wall fractions

The sequential extraction procedure is shown in Figure S8. Freeze-dried algae material was ground with mortar and pestle and extracted two times with acetone-water 70% (V/V), ratio 1:10 (m/V), under constant stirring at 4°C at first for 2 h, and then overnight (21 h) to remove phenolic compounds. After pre-extraction, air-dried algae material was ground and afterwards extracted with double-distilled water (ddH_2_O), ratio 1:10 (w/V), under constant stirring at 4°C overnight. Following centrifugation (4200 *g* at 4°C for 30 min), the precipitate was dissolved in ddH_2_O and freeze-dried. For linkage-type analysis (see below), AE was further purified by tangential cross-flow filtration (MWCO 50 kDa).

The water extracted plant material of *S. pratensis* and *N. obtusa,* respectively, was subsequentially treated with 0.2 mol L^−1^ ammonium oxalate, 0.01 mol L^−1^ hydrochloric acid, 3% (w/V) sodium carbonate and 2 mol L^−1^ potassium hydroxide according to Figure S8. The treatment was performed with each solution in a ratio of 1:100 (w/V) under constant stirring at 70°C for 21 h. Following the sodium carbonate extraction, precipitation with acetone at a final concentration of 80% (v/v) was performed and the precipitate was dissolved afterwards in ddH_2_O. Between the steps, the extract was separated from the insoluble residue by centrifugation at 19 000 g for 30 min. All extracts were dialysed for 3 days against demineralized water (MWCO 12–14 kDa).

Freeze-dried WF was further fractionated using βGlcY, as synthesized in the Classen lab according to Yariv et al. (1962) following the methodology of Classen et al. (2005). The precipitated βGlcY-glycoprotein complex was treated with sodium hydrosulfite at 50°C until the red colour disappeared to release the bound glycoproteins. Afterwards, dialysis was performed for 3 days against demineralized water (at 4°C, MWCO 12–14 kDa) and, finally, the YF was freeze-dried.

### Quantification of monosaccharides

For analysis of neutral monosaccharide composition, the samples were hydrolysed with 2.0 mol L^−1^ trifluoroacetic acid at 121°C for 1 h. After evaporation of trifluoroacetic acid, monosaccharides were converted to their corresponding alditol acetates by reduction and acetylation as described by Blakeney et al. (1983). Then, 50 µL aqueous *myo*-inositol was used as an internal standard at the concentration 10 g L^−1^. For gas chromatography (GC), a system with mass spectrometry detection (MSD) and flame ionization detection (FID) for identification and quantification of neutral monosaccharides was used (GC + FID; model 7890B; MS: model 5977B MSD; Agilent Technologies, Santa Clara, CA, USA; column: Optima-225; Macherey-Nagel, Düren, Germany; 25 m, 250 µm, 0.25 µm; helium flow rate: 1 mL min^−1^; constant temperature 230°C; split ratio 30:1). For clear separation of overlapping peaks especially in the regions of methylated monosaccharides, a linear gradient was introduced in the methodology (initial temperature 200°C, followed by a hold time of 3 min; subsequentially a gradient of 2°C min^−1^ was applied until 243°C was reached). For identification of 3*O*Me-Gal, the validated GC-MS procedure of Pfeifer and Classen (2020) was applied. The content of UA was determined photometrically according to Blumenkrantz and Asboe-Hansen (1973).

### Reduction and labelling of UA with sodium borodeuteride (NaBD_4_)

YF and partial hydrolysed YF samples were carboxy-reduced and labelled with NaBD_4_. Therefore, *N*-cyclohexyl-*N′*-(2-morpholinoethyl) carbodiimide-methyl-*p*-toluenesulfonate and solutions of NaBD_4_ were used as described by Taylor and Conrad (1972). Afterwards the samples were dialysed against demineralized water (at 4°C, 6–8 kDa MWCO) for 3 days and finally freeze-dried.

### Partial degradation of YF

For mild degradation of YF samples, the protocol of Gleeson and Clarke (1979) was used. Samples were hydrolysed in 12.5 mmol L^−1^ aqueous oxalic acid and heated for 5 h at 100°C (Wheaton® V-Vial; Bioblock Scientific, Thermolyne Corp., Dubuque, IA, USA).

### Linkage type analysis

For structural analysis a methodology based on Harris et al. (1984) was used. In the first step, potassium hydride (KH) carbanions were prepared to develop polyalkoxide ions. The sample (1–5 mg of freeze-dried material) was treated with KH in DMSO (0.08 g mL^−1^ KH) and methylated by the addition of 250 µL of methyl iodide. The methylated sample was dissolved in dichloromethane, washed with water three times and hydrolysed with trifluoroacetic acid (2.0 mol L^−1^) at 121°C for 1 h. The permethylated monosaccharides were reduced with 0.5 mol L^−1^ sodium-borohydride in ammonia (2.0 mol L^−1^) at 60°C for 1 h and the reaction was stopped by addition of acetone. Acetylation of the sample was performed by addition of 200 µL of glacial acid, 1.0 mL of ethyl acetate, 3.0 mL of acetic anhydride and 100 µL of perchloric acid. Addition of demineralized water and 100 µL methylimidazole stopped the reaction. After extraction with dichloromethane, the permethylated alditol acetates were separated and detected by GLC-mass spectroscopy as described above (instrumentation as stated above; column: Optima-1701, 25 m, 250 µm, 0.25 µm; helium flow rate: 1 mL min^−1^; initial temperature: 170°C; hold time 1: 2 min; rate 1: 1°C min^−1^ until 210°C was reached; rate 2: 30°C min^−1^ until 250°C was reached; hold time 2: 10 min).

### Determination of protein and Hyp content

Nitrogen content was determined by elemental analysis in the Chemistry Department of CAU Kiel University, Kiel, Germany (HEKAtech CHNS Analyser); protein content was calculated according to the Kjeldahl method (factor 6.25).

Hyp content of all fractions of *Nitellopsis,* as well as YF, partial hydrolysed YF, KOH and insoluble residue of *Spirogyra* samples, was determined colorimetrically as described by Stegemann and Stalder (1967). Calibration was performed by linear regression using a 4-hydroxy-l-proline standard.

### Gel-diffusion assay with βGlcY

Interaction with βGlcY was checked by a gel diffusion assay. An agarose gel (10 mM Tris-HCl, 1 mmol L^−1^ CaCl_2_, 0.9% NaCl, 1% agarose) with six cavities was prepared. Three cavities were filled with 20 µL of βGlcY solution (1 mg mL^−1^ in ddH_2_O) and the corresponding three cavities were filled with sample solutions of *Nitellopsis* and *Spirogyra* AE (100 µg mL^−1^), as well as *Echinacea purpurea* AGP (10 µg mL^−1^) as positive control.

### Elisa

Binding of four different monoclonal AGP-antibodies (JIM13, LM2, LM6 and KM1) against AE, YF and partial hydrolysed YF was tested in an indirect ELISA according to Pfeifer et al. (2020) with sample concentrations of between 2.5 and 100 µg mL^−1^ and antibody dilutions of 1:20 in PBS. For analysis of RG-I epitopes, the antibodies INRA-RU1 and -2 (Ralet et al., 2010) were used at a concentration of 1:200 to analyse YF in comparison to degraded citrus pectin (Nangia-Makker et al., 2002).

### Microscopy

*Spirogyra pratensis* was incubated for 2–3 h in 1 mL of a solution of βGlcY (1 mg mL^−1^ in 0.15 mol L^−1^ sodium chloride). Afterwards, the algae filaments were washed three times with 1 mL of 0.15 mol L^−1^ sodium chloride solution until the reddish colour disappeared. The same procedure was performed as a negative control with the non-reactive αGalY. Small spots of algae tissues were picked with forceps, placed on a glass slide with cover glass and investigated by light microscopy (Carl Zeiss AG, Oberkochen, Germany). Images were acquired using an EOS 1000D digital camera (Canon Inc., Tokyo, Japan).

The field sample of *Spirogyra* sp. was stained accordingly and investigated using a Zeiss Axiovert 200M light microscope (Carl Zeiss AG, Jena, Germany), equipped with an Axiocam HRc camera and axiovision (Carl Zeiss AG).

## Supplementary Material

Table S1-S3, Fig. S1-S6

## Figures and Tables

**Table 1:** Neutral monosaccharide composition of aqueous extracts (AE) from *Nitellopsis obtusa* and *Spirogyra pratensis* (% mol/mol)

Neutral monosaccharide	*Nitellopsis obtusa* AE (*n* = 3)	*Nitellopsis obtusa* AE_purified_ (*n* = 3)	*Spirogyra pratensis* 1 AE (*n* = 3)	*Spirogyra pratensis* 2 AE (*n* = 2)
2-*O*-Me-Rha	–	–	0.2 ± 0.1	0.2 ± 0.0
3-*O*-Me-Rha	–	–	0.9 ± 0.1	1.4 ± 0.7
Rha	18.0 ± 0.4	20.3 ± 0.8	50.4 ± 0.6	45.3 ± 2.9
Fuc	5.8 ± 0	6.9 ± 0.3	7.6 ± 0.0	8.2 ± 0.2
Ara	20.8 ± 0.7	23.6 ± 0.8	5.2 ± 0.4	4.8 ± 0.9
Xyl	3.7 ± 0.5	4.4 ± 0.2	2.1 ± 0.3	3.3 ± 0.8
Man	7.3 ± 0.4	7.6 ± 0.2	0.9 ± 0.1	1.1 ± 0.2
3-*O*-Me-Gal	–	- ± 0.1	0.5 ± 0.1	0.4 ± 0.1
Gal	18.6 ± 0.2	21.3 ± 0.2	29.4 ± 0.6	31.4 ± 1.1
Glc	25.8 ± 0.9	16.0 ± 0.4	2.7 ± 0.1	3.7 ± 0.3

**Table 2:** Neutral monosaccharide composition of Yariv precipitation (YF) from *Spirogyra pratensis* before (YF) and after reduction of uronic acids (YF_UR, %, mol/mol)

Neutral monosaccharide	YF			YF_UR	
	*Spirogyra pratensis* 1 (*n* = 3)	*Spirogyra pratensis* 2 (*n* = 2)		*Spirogyra pratensis* 1 (*n* = 2)	*Spirogyra pratensis* 2 (*n* = 2)
2-*O*-Me-Rha	1.3 ± 0.0	1.0 ± 0.1		1.9 ± 1,3	1.0 ± 0.1
3-*O*-Me-Rha	1.5 ± 0.2	1.3 ± 0.0		1.3 ± 0.6	1.2 ± 0.0
Rha	35.5 ± 2.3	26.3 ± 1.5		27.1 ± 4.0	26.4 ± 1.6
Fuc	5.3 ± 0.1	4.6 ± 0.2		4.8 ± 0.2	5.5 ± 1.5
Ara	3.9 ± 0.3	3.3 ± 0.9		5.4 ± 2.9	2.2 ± 0.1
Xyl	1.3 ± 1.0	1.4 ± 1.0		2.2 ± 2.1	0.8 ± 0.1
Man	1.1 ± 0.1	1.7 ± 0.1		1.0 ± 0.3	1.5 ± 0.2
3-*O*-Me-Gal	1.6 ± 0.3	1.3 ± 0.2		1.6 ± 0.7	1.0 ± 0.0
4-*O*-Me-GlcA*	–	–		3.1 ± 0.1	2.5 ± 0.1
Gal	46.1 ± 0.5	57.5 ± 1.0		46.8 ± 3.4	52.3 ± 2.7
Glc/ GlcA*	2.1 ± 0.2	1.6 ± 0.3		4.9 ± 0.5	5.6 ± 0.0

* Uronic acids not detectable in YF as a result of the method used. Glc/GlcA combines Glc and deuterium-labelled Glc originating from GlcA.

**Table 3:** Linkage type analysis of *Spirogyra pratensis* Yariv precipitation (YF) after reduction of uronic acids before (YF_UR) and after partial acid hydrolysis (YF_UR_hydr_, % mol/mol)

Monosaccharide	Linkage type	*Spirogyra pratensis* YF_UR	*Spirogyra pratensis* YF_UR_hydr_
Ara	1-Ara*f*	1.0	–
	1,2-Ara*f*	tr*	–
Rha	1-Rhap	24.7	22.5
	1,3-Rha*p*	18.5	21.0
Gal	1-Gal*p*	–	7.4
	1,3-Gal*p*	9.1	11.4
	1,6-Gal*p*	5.4	8.6
	1,3,6-Gal*p*	28.4	23.2
Others	1-Fuc*p*	2.6	–
	1-GlcA*p*	2.8	–
	1,4-Glc*p*/GlcA*p*	4.6	3.2
	1,6-Man*p*	2.9	2.7

* tr: below 1%.

**Table 4:** Summary of different arabinogalactan-protein (AGP) features detected in aqueous extracts from *Nitellopsis obtusa* and Yariv precipitation from *Spirogyra pratensis*

Feature of AGP	*Nitellopsis obtusa*	*Spirogyra pratensis*
Precipitation or staining with βGlcY	–	✔
Hydroxyproline	–	✔
Arabinogalactan moiety		
Gal	✔	✔
Ara	✔	–
Reactivity with antibodies against AG epitopes		
JIM13	–	✔
KM1	✔	–
LM6	✔	–

## Data Availability

All relevant data can be found within the article itself and its supporting materials.
