# Supplementary material for: Search for evolutionary roots of land plant arabinogalactan-proteins in charophytes: presence of a rhamnogalactan-protein in Spirogyra pratensis (Zygnematophyceae)
Source: Plant J. Author manuscript; Available in PMC 2022 Mar 21. (PMC7612518; doi:10.1111/tpj.15577)
Supplement: Table S1-S3, Fig. S1-S6 [file EMS143826-supplement-Table_S1_S3__Fig__S1_S6.pdf]

Supplement:

Search for evolutionary ancestors of land plant arabinogalactan-proteins in charophytes: presence of a rhamnogalactan-protein in *Spirogyra pratensis* (Zygnematophyceae)

Lukas Pfeifer[1+], Jon Utermöhlen[1+], Kathrin Happ[1], Charlotte Permann[2], Andreas Holzinger[2], Klaus von Schwartzenberg[3] and Birgit Classen[1*]

[1] Pharmaceutical Institute, Department of Pharmaceutical Biology, Christian-Albrechts-University of Kiel, 24118 Kiel, Germany

[2] Department of Botany, Functional Plant Biology, University of Innsbruck, 6020 Innsbruck, Austria

[3] Biocenter Klein Flottbek, University of Hamburg, 22609 Hamburg, Germany

[+]Both authors contributed equally to the publication.

*Author for correspondence:

Birgit Classen, Pharmaceutical Institute, Department of Pharmaceutical Biology, Christian-Albrechts-University of Kiel, Gutenbergstr. 76, 24118 Kiel, Germany

Phone: +49-431-8801130

e-mail: *bclassen@pharmazie.uni-kiel.de*

Table S1a. Neutral monosaccharide composition of the extracts from *N. obtusa* (% mol/mol).

Neutral monosaccharide	*N. obtusa* $(NH_4)_2C_2O_4$ (n=3)	*N. obtusa* HCl (n=3)	*N. obtusa* Na_2CO_3 (n=3)	*N. obtusa* KOH (n=3)
Rha	14.9 ± 0.5	5.2 ± 1.3	5.7 ± 0.3	1.2 ± 0.1
Fuc	6.5 ± 0.4	3.9 ± 1.0	5.5 ± 0.6	2.1 ± 0.3
Ara	17.5 ± 1.0	7.0 ± 2.1	6.1 ± 0.4	5.8 ± 0.5
Xyl	12.2 ± 0.3	4.5 ± 1.1	5.8 ± 0.4	29.0 ± 1.7
Man	6.9 ± 0.2	4.4 ± 1.4	5.8 ± 0.1	8.3 ± 1.1
Gal	16.8 ± 1.0	6.4 ± 1.9	7.2 ± 0.4	1.4 ± 0.1
Glc	25.2 ± 3.1	68.6 ± 8.9	63.8 ± 2.0	52.2 ± 1.0

Table S1b. Colorimetric determination of uronic acids in the extracts from *N. obtusa* (% w/w).

	N. obtusa $(NH_4)_2C_2O_4$ (n=2)	*N. obtusa* HCl (n=2)	*N. obtusa* Na_2CO_3 (n=2)	*N. obtusa* KOH (n=2)
Uronic acids	18.2 ± 4.9	31.7 ± 2.8	13.4 ± 0.2	5.7 ± 0.3

Table S1c. Neutral monosaccharide composition of the extracts from *S. pratensis* (% mol/mol).

Neutral monosaccharide	*S. pratensis* $(NH_4)_2C_2O_4$ (n=3)	*S. pratensis* HCl (n=3)	*S. pratensis* Na_2CO_3 (n=3)	*S. pratensis* KOH (n=3)
Rha	30.5 ± 1.3	9.3 ± 0.8	14.8 ± 0.2	5.9 ± 0.5
Fuc	10.6 ± 0.1	2.9 ± 1.2	9.5 ± 0.8	8.2 ± 1.0
Ara	6.2 ± 0.3	2.0 ± 0.5	3.2 ± 0.2	1.4 ± 0.2
Xyl	2.9 ± 0.1	4.7 ± 2.7	9.3 ± 0.8	20.8 ± 1.4
Man	1.5 ± 0.1	0.8 ± 0.7	0.9 ± 0.2	2.2 ± 0.9
Gal	20.7 ± 0.3	12.5 ± 3.9	21.2 ± 0.5	30.7 ± 0.7
Glc	27.6 ± 0.3	68.0 ± 8.4	41.1 ± 0.3	30.7 ± 0.9

Table S1d. Colorimetric determination of uronic acids in the extracts from *S. pratensis* (% w/w).

	S. pratensis $(NH_4)_2C_2O_4$ (n=2)	*S. pratensis* HCl (n=2)	*S. pratensis* Na_2CO_3 (n=2)	*S. pratensis* KOH (n=2)
Uronic acids	4.8 ± 0.5	3.3 ± 0.7	7.4 ± 1.1	0.7 ± 0.3

Table S2. Linkage type analysis of *Nitellopsis obtusa* $AE_{purified}$ (% mol/mol).

monosaccharide	linkage type	*N. obtusa* $AE_{purified}$
Ara	1-Ara*f*	15.8
	1,5-Ara*f*	14.6
Rha	1-Rha*p*	tr*
	1,4-Rha*p*	11.9
	1,2,4-Rha*p*	2.4
	1,3,4-Rha*p*/Fuc*p*	5.9
Gal	1-Gal*p*	tr*
	1,3-Gal*p*	13.5
	1,6-Gal*p*	12.1
	1,3,4-Gal*p*	2.2
	1,3,6-Gal*p*	11.3
	1,4,6-Gal*p*	2.2
others	1-Glc*p*	1.6
	1,4-Glc*p*	3.9
	1,3,4-Glc*p*	2.6

*tr: below 1%

Table S3. Changes of galactose linkage types of *S. pratensis* YF before and after partial acid hydrolysis (% mol/mol).

monosaccharide	linkage type	*S. pratensis* YF_UR	*S. pratensis* YF_UR_{hydr}
Gal	1-Gal*p*	-	14.6
	1,3-Gal*p*	21.2	22.5
	1,6-Gal*p*	12.5	17.0
	1,3,6-Gal*p*	66.3	45.8

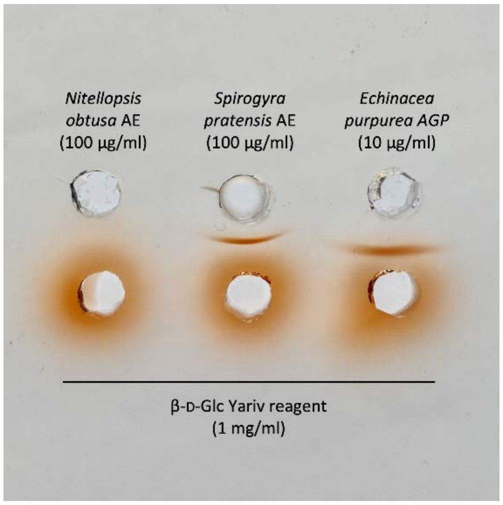

Figure S1. Gel diffusion assay with βGlcY and aqueous fractions from *N. obtusa* and *S. pratensis*. The precipitation band indicates presence of AGPs. AGP from *Echinacea purpurea* was used as positive control.

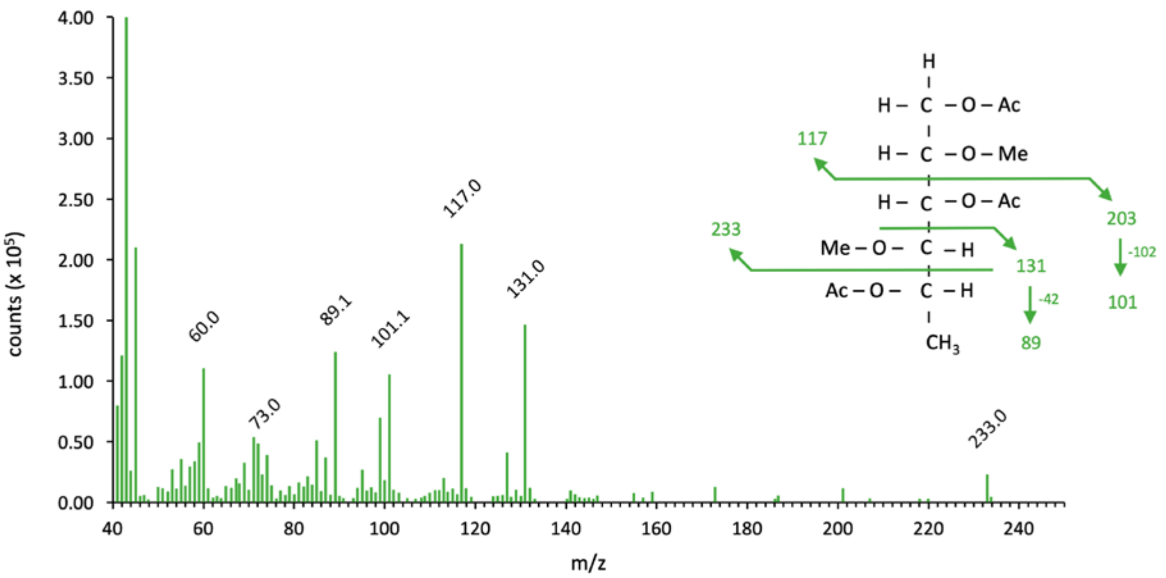

Figure S2. Mass spectrum of partially methylated alditol-acetate (PMAA) derived from 1,3-linked Rha*p* in YF from *S. pratensis.*

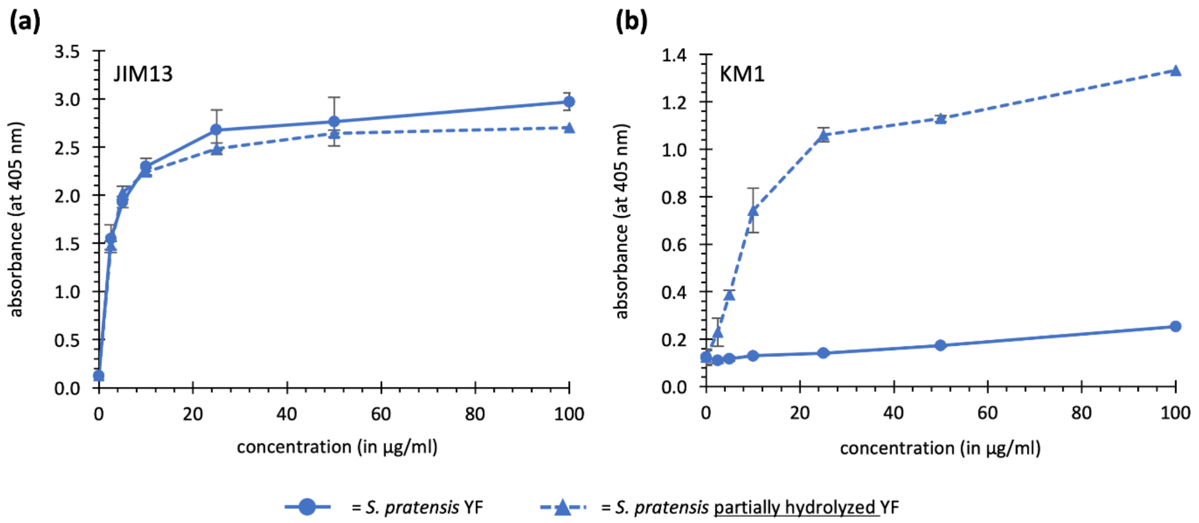

Figure S3. Reactivity of *S. pratensis* YF before and after partial mild acid hydrolysis with antibodies directed against AGP glycans in ELISA. (a) JIM 13. (b) KM1.

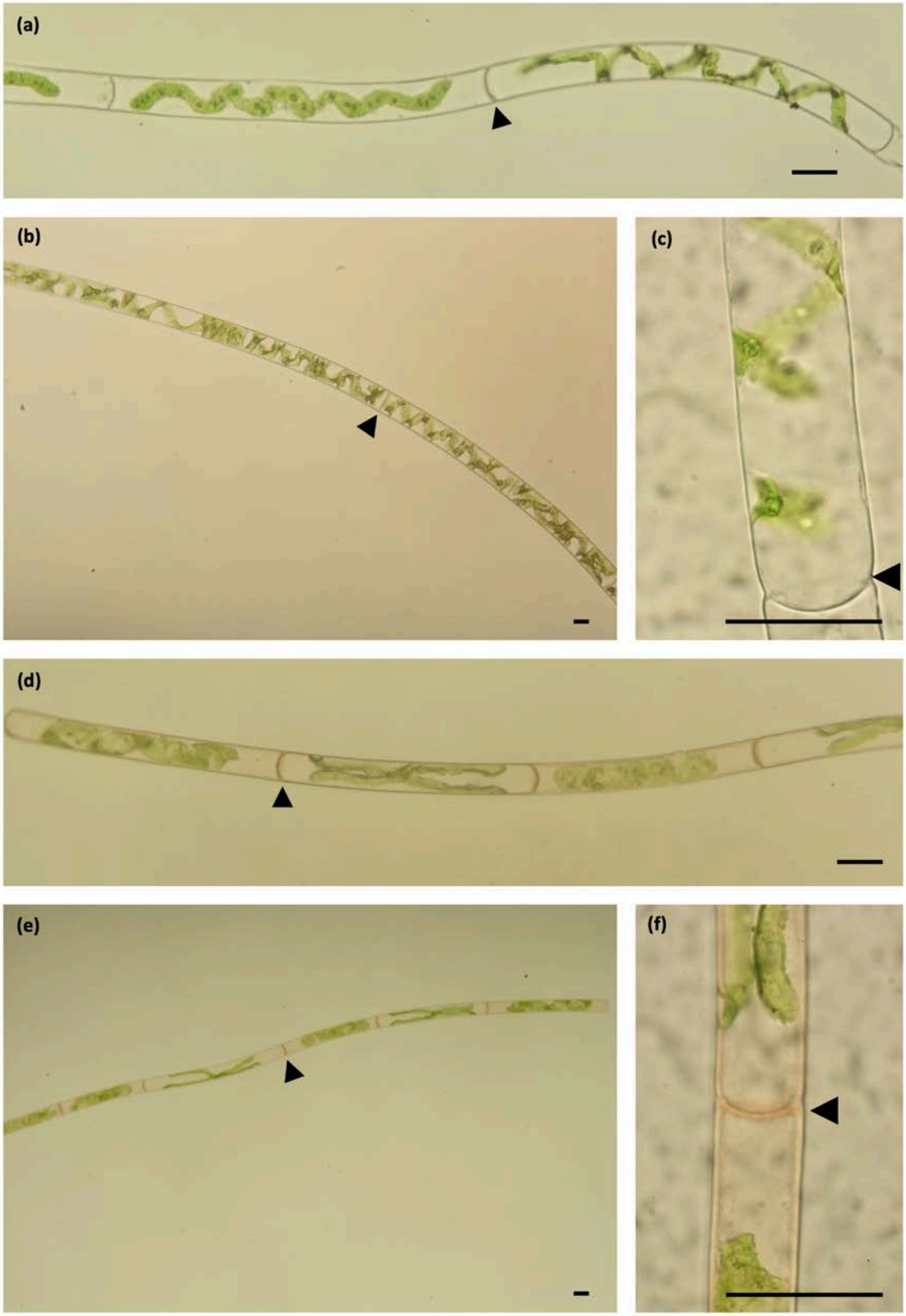

Figure S4. Culture of *Spirogyra pratensis* with vegetative growth.
(a-c) Staining with αGalY (negative control). Arrows show transverse walls, which stay unstained with αGalY.
(d-f) Staining with βGlcY, which binds to RGPs. Arrows show transverse walls, which are stained with βGlcY, indicating enrichment in RGPs.
Scale bars 20 μm.

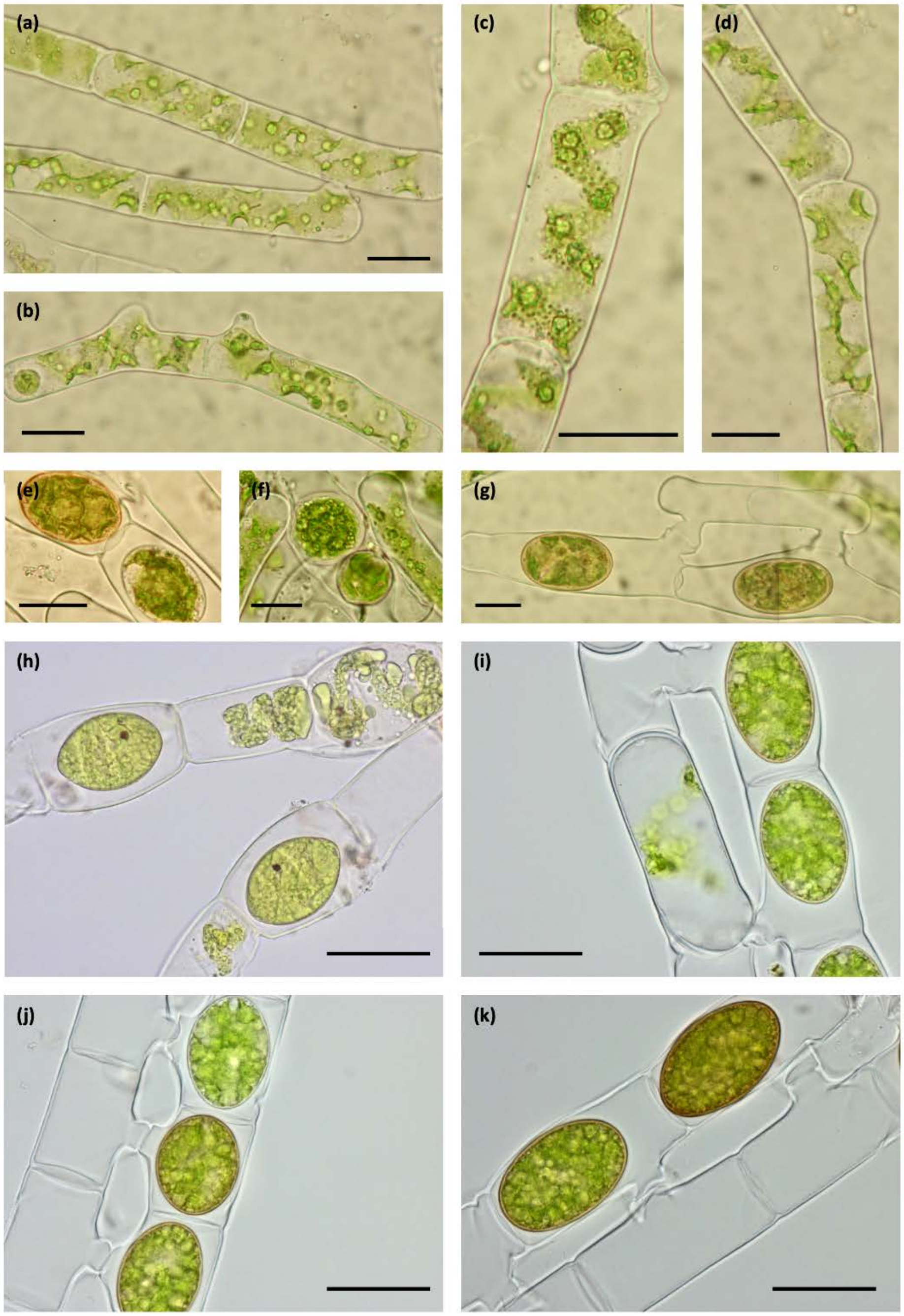

Figure S5. Conjugation and zygospores of cultured *Spirogyra pratensis* and *Spirgyra* sp. field sample from Kühtai, Tyrol, Austria.

(a-g) *Spirogyra pratensis*, staining with αGalY=negative control.

(h) *Spirogyra* sp., staining with αGalY=negative control.

(i-k) *Spirogyra* sp., without staining.

(a,b) Early stages of scalariform conjugation. (c,d) Early stages of lateral conjugation.

(e-k) Stages with zygospores, younger zygospore walls colourless, older ones with red-coloured walls.

Scale bars 20 µm.

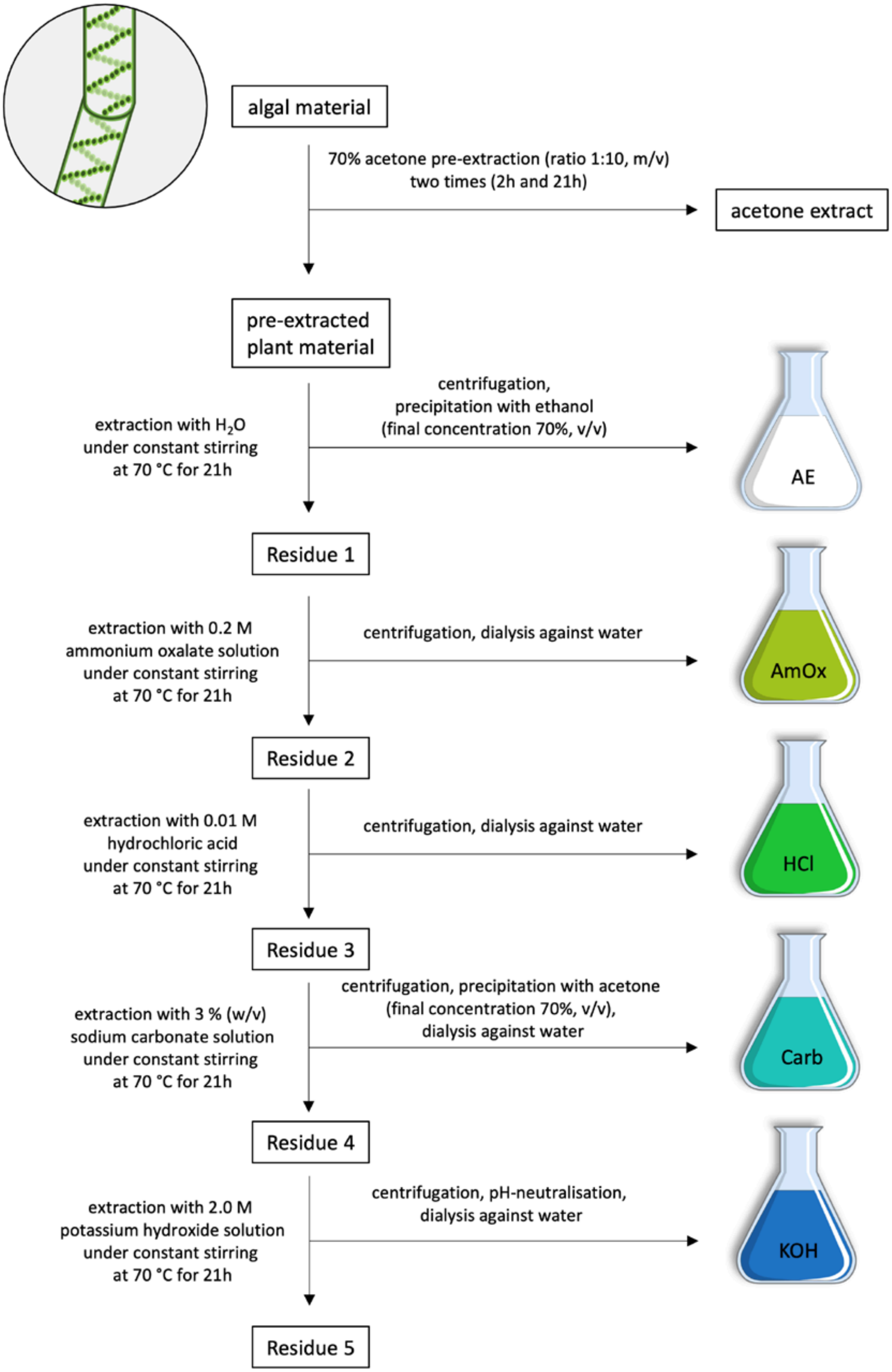

Figure S6. Schematic presentation of extraction procedure for *Nitellopsis* and *Spirogyra*.
